# Mamas in Harmony: protocol for a pilot RCT and process evaluation of a music and social support intervention establishing the feasibility of reducing anxiety and stress in postnatal mothers

**DOI:** 10.1186/s40814-025-01629-1

**Published:** 2025-04-10

**Authors:** Corinna A. Colella, Jenny McNeill, Una McCann, Fiona A. Lynn

**Affiliations:** School of Nursing and Midwifery, Queen’s University Belfast, Belfast, UK

**Keywords:** Music, Anxiety, Stress, Mothers, Infant, Postnatal, Feasibility, Randomised controlled trial

## Abstract

**Background:**

Postnatal mothers can experience anxiety, stress and difficulty with mother-infant attachment with potential to lead to an anxiety disorder/depression. There has been an increase in interest in non-pharmacological interventions involving music, with limited evidence assessing effectiveness in this population within randomised controlled trials (RCTs). Mamas in Harmony is a novel music/social support intervention. The objective is to assess the feasibility/acceptability of conducting a full RCT to test the effect and cost-effectiveness of Mamas in Harmony compared with usual care.

**Methods:**

The pilot RCT aims to recruit 60 mother-infant dyads through social media, community organisations and stakeholder networks, randomly allocated on a 2:1 ratio to the intervention and control group. The intervention group will receive eight 1 h weekly Mamas in Harmony sessions plus usual care, and the control group will receive usual care only. Feasibility measures include recruitment, retention and attendance at intervention sessions. The process evaluation aims to establish acceptability of the intervention involving survey evaluation and semi-structured interviews. A priori guidelines have been agreed to establish criteria for progression to a definitive RCT.

**Discussion:**

The current evidence supports the use of music in reducing anxiety and stress with the recommendation for inclusion of social support. This study has potential to provide robust evidence of the feasibility and acceptability of a music and social support mother-infant group intervention in preparation for a future definitive trial, should progression criteria be met.

**Trial registration:**

ClinicalTrials.gov ID: NCT05930990, registered retrospectively on 5 July 2023. Recruitment commenced on 16 March 2023.

**Protocol version:**

30 October 2023 Vn 1.0

**Supplementary Information:**

The online version contains supplementary material available at 10.1186/s40814-025-01629-1.

## Background

Poor postnatal mental health has the potential to lead to negative outcomes for both mother and child and is a current leading public health issue with significant economic burden if untreated [1]. Symptoms of low mood, anxiety, stress, and/or feeling isolated may be experienced by around 15–20% of mothers in the first year after childbirth [2]. If left undiagnosed/untreated, symptoms may progress to an anxiety disorder and/or depression [3]. Recent research shows postnatal depression is significantly higher in women 6 months after giving birth during the Covid- 19 pandemic compared to pre-pandemic levels when comparing data from maternity surveys completed in England in 2014, 2018 and 2020 [4].

Studies investigating the effect of poor postnatal mental health have found an adverse impact on mother-infant attachment [5], bonding, self-regulation and empathy [6]. Consequently, mothers may find it difficult to engage with their infants both emotionally and behaviourally resulting in reducing their physical contact [7]. Provision by the mother of a less-stimulating environment, being less attuned to their infant [8] and feelings of reduced competence in the mother [9] have also been reported.

The postnatal period is considered by the National Institute for Health and Care Excellence (NICE) to last for 1 year after the birth of the child [10]. Prevalence of an anxiety disorder across pregnancy and the postnatal period has been estimated to be 20.7% [11]; however, the literature remains less established and less understood for postnatal anxiety compared to postnatal depression [12]. Despite screening, postnatal mental health problems often go unidentified, undiagnosed and untreated for many women, or they do not meet the eligibility threshold for specialist mental health services after the birth of their baby [13]. Women may fear stigma and negative perceptions if they disclose a mental health problem in pregnancy or the postnatal period [13]. Current pharmacological treatment options offered by health professionals involved in the care of mothers often have potential for side effects and low uptake and adherence, especially for breastfeeding mothers [14]. Psychotherapy has presented challenges with mixed results of effect and short-lived improvements [15, 16].

The economic cost of poor mental health for mothers has also been considered. The NHS Long-Term Plan [10] identified that perinatal (pregnancy and postnatal period combined) anxiety costs approximately £35,000 per case, of which £14,000 relates to the impact on the child. Therefore, introducing low-cost, non-pharmacological interventions that target anxiety and provide social support to mothers may be worthwhile and potentially cost saving for the NHS, families and society as a whole.

The field of arts in health is rapidly expanding with evidence from research into arts-based initiatives in primary and secondary health care. The findings of seven experimental studies synthesised within a systematic review have shown a variety of benefits of singing among 327 participants living with mental health conditions. Participants across the UK and Australia demonstrated improvements in depression, quality of life and mental wellbeing following singing interventions lasting at least 8 weeks [17]. Six qualitative studies further indicated that group singing resulted in a sense of belonging, improved self-confidence and were considered enjoyable [17].

A recent systematic review assessed the effect of mother-infant group music sessions (any type of music as the primary component) on maternal postnatal depression [18]. The review identified two studies conducted in the UK and in Australia, both measuring maternal depression following 10-weekly music sessions for the intervention group compared to no intervention for the control group [19, 20]. No clear theoretical underpinnings were detailed within either study. While the pooled estimate of effect favoured the music intervention when compared to the control group, it was not statistically significantly different (*SMD* − 1.61: 95% *CI* − 4.09, 0.87) [18]. No statistical difference was found in total parenting stress scores post-intervention between groups [20]. However, secondary outcomes were statistically significantly different, with anxiety and stress reduced and maternal self-efficacy improved in the intervention group when compared to control post-intervention [18]. Findings from this review suggested a potential for music-based mother-infant group interventions to improve maternal anxiety, stress and self-efficacy. Qualitative research conducted alongside one of the studies showed mothers found singing facilitated social cohesion [21]. A subsequent process evaluation [22] of this singing intervention found postnatal mothers recommended the opportunity for coffee after each session to allow for socialising with other mothers [22]. Further recommendations from the studies were a larger space so mothers had room to leave the immediate circle of the activity if required [22] and a shorter format from the 10-week duration due to only 44% of participants attending ≥ 5 sessions [20]. More recently, a single-arm feasibility study has shown acceptability of an online singing intervention for mothers supporting their mental health [23]. An in-person group singing intervention, “Music and Motherhood”, has also been explored in terms of feasibility study design [24]. The brief research report concluded that such an arts in health. adaptation from a UK setting and implemented in Denmark and Romania using a single-arm feasibility study design [24]. The brief research report concluded that such an arts in health intervention could be adapted to different sociocultural contexts when delivered in a way that was sensitive to the culture of the population [24].

### Rationale

In the context of the available evidence, a music-based group intervention for mother-infant dyads may have the potential to reduce symptoms of anxiety and stress, promote social support and target an improvement in the mother-infant relationship [25]. This study will seek to test a novel mother-infant group intervention that combines music and social support in a feasibility study with a pilot randomised controlled trial, in order to provide a foundation from which a protocol for a definitive RCT could be developed. The feasibility of a full economic evaluation will also be evaluated to establish best methods for collecting data and a process evaluation completed to understand the mothers and intervention facilitator/s experiences of the intervention. The SPIRIT 2013 Statement recommendations have guided the creation of this protocol [26], in conjunction with additional guidance from the CONSORT extension to pilot and feasibility trials [27] to ensure transparency and quality of reporting.

### Objectives for pilot RCT

1. Conduct pilot RCT of a music-based intervention (Mamas in Harmony) alongside usual care versus usual care only to establish the feasibility of a definitive RCT in terms of recruitment rate, retention rate, adherence to the intervention and completion rates of outcome measures.
2. Assess feasibility of conducting a full economic evaluation in terms of completion rates of data collection tools and assess promise of cost-effectiveness.
3. Use data obtained to assist with review and adjustment of the protocol, intervention and creation of a logic model.

## Methods

### Trial design

The pilot RCT is a parallel two-group study design and an economic evaluation. Block randomisation will be carried out on a 2:1 allocation. A process evaluation will be conducted alongside consisting of a survey and semi-structured interviews.

### Study setting

The pilot RCT will be carried out in Belfast, a city in Northern Ireland. The intervention setting is a community venue in Belfast and will be a single study site.

## Pilot RCT

### Eligibility criteria

The focus of this study is not on effect size, and, so, a power calculation has not been estimated. Participants for the pilot RCT will be mothers who are aged 16 or over and who have a baby ≥ 14 days to ≤ 4 months of age at time of consent, and they will be invited to participate. They must be able and willing to provide consent, as identified by having a satisfactory understanding of English and comprehension of the participant information sheet and consent form. Women will not be able to enter the study if they have given birth in the last 14 days, their infant is > 4 months of age at time of consent and/or have identified as not having satisfactory understanding of English and comprehension of the participant information sheet and consent form. Mothers who have received a diagnosis of, or being treated for, severe mental illness including bipolar disorder, psychoses or Schizophrenia within the past 6 months (discovered through self-report at time of eligibility screening) will also be excluded. This is due to the existing evidence supporting the use of singing interventions for symptoms of postnatal anxiety and stress [20] and not as a treatment for severe mental health conditions.

### Recruitment 

**Fig. 1:**
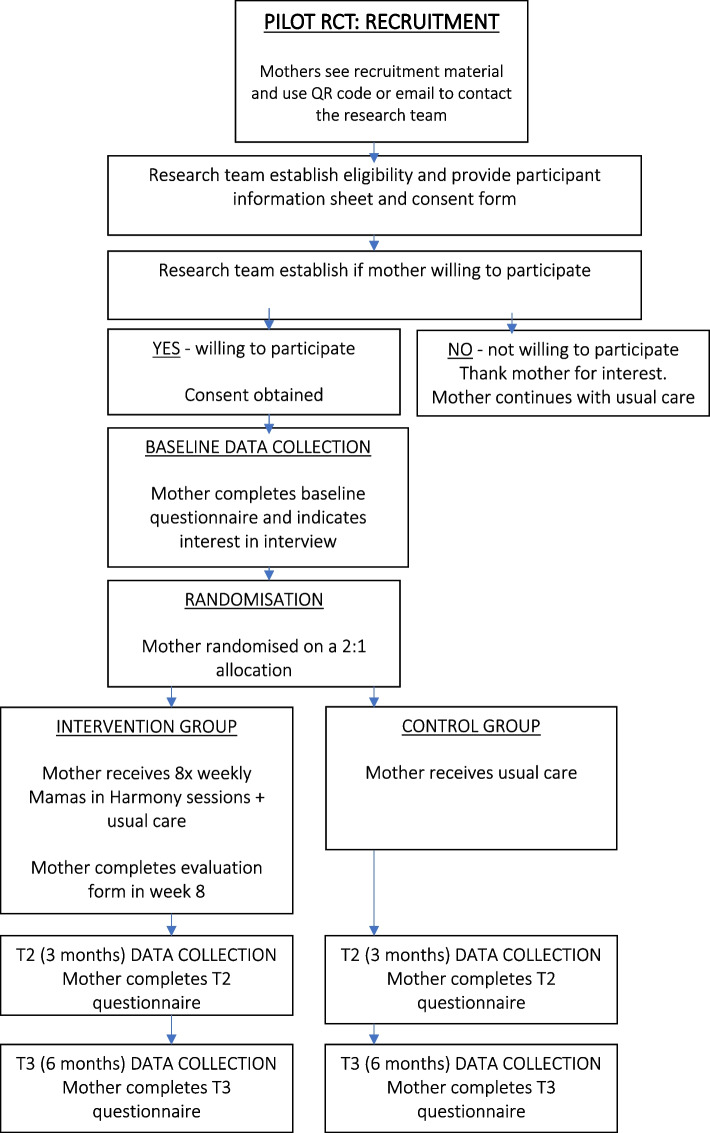
Recruitment flow diagram — pilot RCT

The recruitment strategy to identify and approach potential participants will involve posts on social media, emailing via contact lists of relevant local community organisations and displaying of recruitment posters on communal/public facing noticeboards. Recruitment material will contain an email contact for the researcher and QR code, which when scanned, directs potential participants to the study webpage containing an introduction to the study, a participant information sheet and online contact form. Mothers who express interest in participating will be contacted by the researcher and screened for eligibility. If eligible, they will be invited to participate, informed and, if interested, will provide informed consent, obtained by the researcher (CC). Recruitment will last approximately 1 year. A recruitment flow diagram has been created for the pilot RCT (Fig. 1).

### Randomisation

Following informed consent and baseline data collection, participants will be randomly allocated to the intervention group receiving Mamas in Harmony and usual care or the control group receiving usual care alone. Usual care consists of postnatal care offered by Health and Social Care (HSC) services in Northern Ireland provided by the mother’s community midwife, GP and health visitor. Usual care comprises of mother and infant health and wellbeing checks, infant weighing, infant feeding advice and support, developmental assessments and additional visits based on need and capacity [28].

The sequence generation for randomisation of study participants will be carried out in blocks of 6 with a 2:1 allocation to the intervention and control group using a computer-generated random allocation sequence on Randomization.com (http://www.randomization.com). Randomisation will be completed by a member of the research team (F. L.) who is not involved in the recruitment process to ensure allocation concealment from participants and the researcher recruiting individuals (C. C.).

Owing to the nature of the intervention, it is not possible to blind participants or personnel delivering the intervention to group status following randomisation. As all outcomes will be self-reported by the mother, it will not be possible to blind outcome assessors to group status either.

### Intervention 

The Mamas in Harmony intervention is underpinned by three key theoretical concepts: (i) the biopsychosocial model of health, (ii) self-determination theory and (iii) the psychology of flow. The intervention consists of music and social support delivered during 1-h face-to-face sessions each week for 8 consecutive weeks and is based on a group music activity for mother-infant dyads developed by Una McCann, a singer, songwriter and instrumentalist who currently runs community choirs with up to 200 adults per week in attendance. Her prior experience involves 8 years working with children for the Junior Academy of Music in Northern Ireland as a choir leader and music tutor. The music component of the intervention has been previously tested and delivered to mothers and infants at SureStart facilities across Northern Ireland enabling further development for the current intervention. Following a systematic review of evidence of music interventions [18], which highlighted mothers recommendation for coffee after each session to allow for socialising with other mothers, a social support component was added to the intervention, the duration of sessions set at 8 weeks and a venue chosen which allows mothers space to break away from the immediate circle of activity if required. Approximately, 10–12 mothers allocated to the intervention group will attend the same community venue where the sessions will take place each week with their babies. The intervention involves mothers attending a group setting in a room each week with their babies. They will be invited to sit on upon soft cushioned play mats in a circle formation with their babies lying on blankets in front of them. Small, colourful, age and developmentally appropriate toys and sensory objects will be available for baby to look at and touch. The singing component will last 40 min and begin with welcoming each mother and infant to the group. The facilitator then introduces a mix of songs that are easy to learn and pleasing to listen to without the need for lyric sheets. Mothers will be encouraged to join in singing, feel connected to each other through the song and will involve some gentle bouncing/rocking movements with the baby. Mothers will be encouraged to interact with their baby through various sensory stimuli involving touch, sound and visual, for example tickling fingers, gentle massage and humming sounds close to their baby’s ear. The group session will end with a social support component, with mothers encouraged to stay for refreshments and informal facilitated social support, where conversation with and between mothers will be initiated and supported by the intervention facilitator and assistant. Mothers will be encouraged to interact with other mothers to promote social connection. The TIDieR checklist [24] was used to aid reporting of the intervention.

A strategy to improve adherence to the intervention protocol will be a text message reminder for attendance the day before each weekly session.

### Feasibility measures

The primary measure is rates of recruitment and retention using screening logs.

### Secondary outcomes

Secondary feasibility measures are rate of adherence of participants across each stage of the pilot RCT using study registers, with reasons for nonattendance if disclosed. Also, rate of attendance to intervention sessions, with reasons for nonattendance if disclosed. Completion rate of outcome measurement tools including Generalised Anxiety Disorder- 7 scale (GAD- 7), Edinburgh Postnatal Depression Scale (EPDS), Perceived Stress Scale (PSS), Karitane Parenting Confidence Scale (KPCS), Social Support Survey (SSS), Maternal Postnatal Attachment Scale (MPAS), EuroQol EQ- 5D- 5L, and service and resource use log will also be collected.

The GAD- 7 was developed to assist health professionals in detecting generalised anxiety. While not specific to postnatal mothers, the GAD- 7 scale is currently recommended by NICE [2]. There are no validated tools to aid diagnosis specifically of postnatal anxiety [12]. The GAD- 7 consists of seven questions with a choice of four responses based on how the individual has felt over the past 2 weeks which is measured on a scale of 0–21. Scores of 5, 10 and 15 are taken as the cut-off points for mild, moderate and severe anxiety respectively. When used as a screening tool, further evaluation is recommended when the score is 10 or greater [29]. The EPDS was developed to assist health professionals in detecting postnatal depression. The scale consists of 10 questions with a choice of 4 responses based on how the individual has felt over the past 7 days and is measured on a scale of 0–30. A score of more than 10 suggests depression may be present, and further evaluation is recommended [30]. The PSS was developed to assist in assessing stress level in young people and adults aged 12 and above. It evaluates the degree to which an individual has perceived life as unpredictable, uncontrollable and overloading over the previous month, consisting of 10 questions. The PSS score is obtained by summing across all items. Higher scores indicate higher levels of perceived stress. There are no published score cut-offs as this is not a diagnostic instrument [31]. The KPCS was developed to assist in the support and development of parenting skills for parents of children 0–12 months of age and contains 15 items with scores of 0–45. Parents scoring 39 or less may be experiencing low levels of parenting confidence [32]. The SSS consists of 19 questions across 4 separate social support subscales and an overall functional social support index. A higher score for an individual scale or for the overall support index indicates more support [33]. The MPAS was developed to assess the mother’s emotional response to her infant along a number of dimensions relating to parent-infant attachment and has 19 items with the number of response categories varying between 2 and 5. Item totals are summed to obtain the scale score. Lower scores indicate lower attachment and vice versa [34]. The EuroQol EQ- 5D- 5L asks respondents rate their health-related quality of life today on five dimensions (mobility, self-care, usual activities, pain/discomfort and anxiety/depression) in order to assess treatment effects and investigate gains (or losses) in reported health status [35]. A service and resource use instrument developed and used previously by the research team (F. L., J. M.) for a similar population will be used in this study to capture and cost public sector service and resource use for postnatal mothers. All tools will be administered to assess whether it is an appropriate outcome measure for a full-scale trial, as determined by completion rate and proportion of missing data.

### Sample size

A total sample size of 60 mother/infant dyads has been chosen. Recommended sample sizes for feasibility trials vary from 10 to 75 depending on the objective of the study [36]. As feasibility trials are not hypothesis-driven, we did not perform a power calculation to detect a difference between groups on an outcome of intervention effect. Instead, we estimated our sample size based on the primary outcome of study retention rate for the overall sample. We used the most conservative retention rate reported in previous RCTs involving group music sessions for postnatal mothers [19]. With a recruitment target of *n* = 60, the anticipated retention rate at primary end point of 80% can be estimated to within a 10% margin of error with 95% confidence. Between 10 and 12 mother-infant dyads will participate in a Mamas in Harmony group at a time and then repeated 3–4 times to reach the required sample size receiving the intervention.

### Process evaluation

The process evaluation will seek to understand perspectives on barriers and facilitators to study participation (intervention/control group) and to explore expectations of and satisfaction with the intervention (intervention group). It will also seek to understand perspectives on barriers and facilitators to intervention delivery to determine improvements needed for future research. Finally, the process evaluation will enable understanding of the perspectives on implementation in terms of fidelity (intervention delivered as intended).

### Participants 

Participants for the survey evaluation will be mothers who participated in the study and were part of the intervention group. Participants for the semi-structured interviews will be mothers who participated in the study and were part of the intervention group or control group and who expressed interest in participating at baseline and intervention facilitator/s who delivered at least one Mamas in Harmony session.

### Recruitment

All mothers allocated to the intervention group who attend the final Mamas in Harmony session will be invited to complete a survey evaluation of the intervention. Survey evaluation will take place at the community venue in which intervention delivery took place.

### Selection

Mothers who indicated an interest in taking part in an interview will have their study ID entered into a MS Excel file where a random number generator will be used to compile a random list of all the study IDs. It is anticipated that 12 (20%) of participants will indicate they will be interested in an interview and that on invite, 8 of these will agree, provide consent and be interviewed. In the first instance, the mothers of the first eight randomly generated study IDs will be approached by the researcher through the participant’s preferred mode of contact, informed of this component of the study and invited to participate. Recruitment will continue, by contacting the mother assigned to sequential study IDs on the list, until eight interviews have been completed. The intervention facilitator/s will be invited to participate in an interview following completion of all intervention sessions. Semi-structured interviews will take place online, via Microsoft Teams or face to face as preferred.

### Acceptability measures

The measures used for the process evaluation include exploring expectations of and satisfaction with the intervention and ideas for improvement using evaluation forms. These will be administered to mothers in week 8 at the final session of the intervention. The evaluation form will include a number of questions with possible responses on a scale of 0–10, a higher score indicating a better rating, scale of strongly disagree to strongly agree, scale of very unlikely to very likely, multiple-choice questions and free text answer boxes. The evaluation form will also enable insight on the acceptability of the intervention in terms of being liked or disliked, appropriate, benefits gained, to identify any barriers to attendance and/or engagement that exist and recommendations for improvement. This information will then provide further prompts alongside the interview schedule created for use with a subset of study participants.

The schedule for enrolment, delivery of intervention and assessment of outcomes for both the pilot RCT and process evaluation has been created and illustrated in a Standard Protocol Items: Recommendations for Interventional Trials (SPIRIT) diagram in conjunction with additional guidance from the CONSORT extension to pilot and feasibility trials [27] which can be found in Table 1.

**Table 1:**
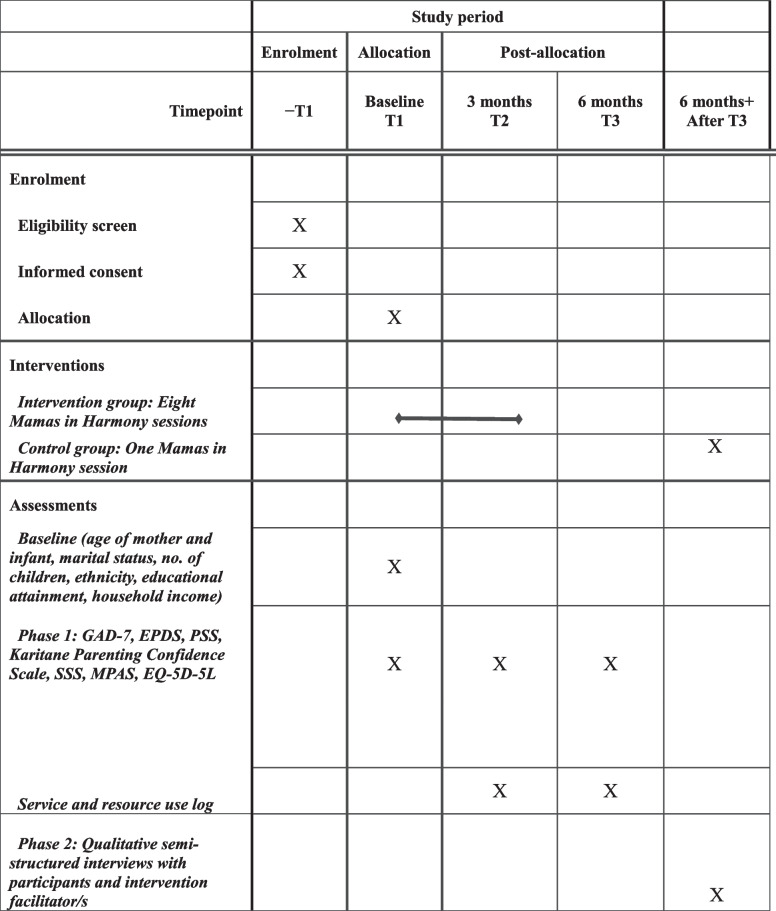
SPIRIT diagram illustrating the schedule of enrolment, implementation of interventions and timeline of assessments

### Data collection

Recruitment, retention, attendance and adherence data will be collected using screening logs and study registers. Outcome assessment tools including GAD- 7, EPDS, PSS, KPCS, SSS, MPAS and EuroQol EQ- 5D- 5L will be collected online using Qualtrics survey platform or via a paper copy as preferred. Service and resource use logs will be completed in paper form.

An email reminder will be sent after 2 weeks to encourage retention and completion of follow-up measures. This will be followed by a second and final email reminder after a further 2 weeks if still no response. A £15 retail gift card will be provided to all participants on completion of the pilot RCT at 6 months.

Evaluation forms will be distributed to participants allocated to the intervention group in week 8 at the final session of the intervention. They will be informed that they do not have to complete the form if they do not wish to, and that the forms are anonymous.

Semi-structured interviews were chosen as the preference over focus groups for participants, and an outline interview schedule has been created. Interviews will enable more flexibility in relation to planning and identification of a day and time suitable to individual participants. Sociodemographic characteristics, including marital status, ethnic group, highest educational attainment and household income of participants, will be collected at the start of the interview to establish a profile of the participants taking part in the interviews.

While interviews are more time-consuming than focus groups for the researcher, they are more convenient to the mother and allow confidentiality for the mother in the discussion and so anticipated to be more likely to have higher participation rates. Interviews are further anticipated to provide rich data in relation to the depth of understanding gained of the mothers’ experiences [37]. Interviews will be conducted and recorded online via Microsoft Teams or face to face as preferred.

For intervention facilitator/s who are approached, informed, invited to participate and who consent, a semi-structured interview will be conducted and recorded upon completion of all cycles of sessions. This will allow them to provide their professional opinion on barriers and facilitators to intervention delivery to determine improvements needed for future research and to understand perspectives on implementation in terms of fidelity (intervention delivered as intended), to inform future research. These will also be conducted and recorded online or face to face as preferred.

Interview schedules for both mothers and intervention facilitator/s have been developed and were informed by current evidence. Questions will also focus on the facilitators/barriers to participation in the intervention and research study to inform future research (see Table 2).

**Table 2:** Examples of interview questions

**Intervention group**
• What did you like most about attending the Mamas in Harmony sessions? Why? (Prompts: specifically ask about singing component and social support component, for example in terms of mood, confidence, social support, attachment/bonding with baby)
• Was there anything you did not like about the Mamas in Harmony sessions? Why? Is there anything that could have been done to help with that?
• Do you feel sessions suited you and your baby? If yes, in what way? (Prompts: what part/s of the session felt relevant/supportive/helpful/calming/nurturing/other description used by mother)
• Do you feel you and your baby have benefited from the Mamas in Harmony sessions? In what way? (Prompts: how you feel about yourself/your baby/your needs, how you feel about singing, the relationship you have with your baby, learned new skills or ideas, being part of a group, opportunity to connect with other mothers)
• Do you feel duration of each session was about right? (Prompt: for music component, for social support component)
• Do you feel the number of sessions offered was enough to be beneficial for you and your baby?
• Do you feel the venue where sessions were held was right? (Prompt: comfortable, enough space, felt able to relax)
**Control group**
• Did you feel you were given sufficient information about the study before you decided to take part?
• Did you complete your questionnaires online (Qualtrics platform) or a paper copy? *If Qualtrics*
o Did you encounter any difficulties? (Prompts: navigating between questions, saving and returning to it at a later date, submitting)
o Did you find the questionnaire layout clear and easy to follow?
o Did you complete all the questions within each questionnaire?
o If not, what was the reason for that? (Prompts: too time -consuming, difficult to understand, information requested was too sensitive)
**Intervention facilitator/s**
• What did you enjoy most about delivering the Mamas in Harmony sessions?
• Is there anything you did not enjoy about delivering Mamas in Harmony sessions?
• What do you think worked well?
• What do you think did not work well?
• Do you feel sessions were tailored and appropriate for mothers and babies?
• Were there any challenges you encountered in delivering Mamas in Harmony sessions? (Prompt: with mums, with babies, with support staff, with the venue, with the intervention materials)

### Data management

All data will be handled in line with General Data Protection Regulation (GPDR) and other relevant regulations [38]. Data collected online via Qualtrics, Microsoft Teams interview recordings or MP4 audio interview recordings will be saved on an encrypted hard drive approved by the research institution and ethics committee. Data collected in paper form will be stored in a lockable filing cabinet behind a locked door on university premises. Only members of the research team will have access to the data.

### Data analysis

For the pilot RCT and surveys within the process evaluation, continuous data will be presented as means and standard deviations and categorical data presented as frequencies and percentages, focusing primarily on rates of recruitment, retention, adherence, attendance, completion of each measurement tool and proportion of missing data. While means and standard deviations will be presented for each outcome measure, the study is not statistically powered to establish effect; therefore, summary scores will not be used for hypothesis testing. However, we will present estimates of the differences between groups and 95% confidence intervals for each outcome measure. Recruitment, participation and retention rates will be reported and presented in a CONSORT flow diagram [39]. Participants who drop out versus retained at 8 weeks (immediately post intervention) and participants who drop out versus retained at 26 weeks (post randomisation) will be reported on in terms of maternal characteristics at baseline for the intervention and control group.

For the process evaluation, audio recordings from the interviews will be transcribed verbatim by the researcher, and pseudonymised transcripts will be uploaded to NVivo and analysed using an interpretive qualitative approach based on the principles of thematic analysis [40]. This type of analysis focuses on identifying themes throughout the data and then be coded by identifying persistent words, phrases, experiences or concepts. Data will then be grouped according to topic, allowing further identification of sub-themes. Following coding, the data will be categorised to reflect the overall picture of the data and relationships between categories. Related categories will be merged into themes [37]. Reflexivity will be practiced throughout the conduct of the pilot RCT including analysis, and individual analysis will be conducted by the research team and then discussion and agreement. Therefore allowing the researcher to aim to understand perspectives from the point of view of the participant. This allows any biases or assumptions about the content to be examined and for the researchers to undergo a process of self-examination [41]. These themes will be used to gain an understanding of the perspectives of mothers and intervention facilitator/s participating in the study.

A data monitoring committee will be established for the full RCT but is not required for the feasibility work proposed.

No significant physical risks are expected to be associated with the participation in this study. A health and safety assessment of the venue for its suitability for mother and infant classes will be conducted prior to commencement of the study and on an ongoing basis during the study. The risk of psychological harm is expected to be minimal. Previous research using a similar intervention has shown no adverse events to study participants [19]. The intervention facilitator and assistant facilitator will be vigilant during delivery of the intervention, process evaluation interviews and during any other contacts with study participants for any sign of distress observed or verbalised by the mother. If this occurs, the researcher will take appropriate action as set out in the distress protocols for both the Mamas in Harmony sessions and interviews.

It is acknowledged that outcome data provided at post-intervention and follow-up timepoints by mothers during the study would not normally be analysed until completion. However, due to completion of outcome measurement tools for anxiety and depression (GAD- 7 and EPDS) having the potential to identify scores above the recognised clinical threshold for the tool representing significant symptoms and/or risk to the mother and infant, these data will be analysed by the researcher within 1 week of receipt of the completed questionnaire from the mother. This will enable the researcher to signpost the mother to her GP and other appropriate supports as per the distress protocol.

Once follow-up is complete and participants have exited the study, the control group will be offered one Mamas in Harmony session to enable them to have experience of the intervention.

### Progression to definitive RCT

Due to the nature of the study, it is not powered to test hypotheses related to the effect of the intervention in comparison to usual care. Instead, it has been designed to support development and assess feasibility of conducting a future definitive RCT [27]. For progression to a definitive RCT, the following a priori guidelines will be used: mother/infant dyads are recruited within a 6-week timeframe, Mamas in Harmony classes have a minimum of 10 mother/infant dyads allocated to each group of classes in the recruitment timeframe, rate of attendance in Mamas in Harmony classes is ≥ 70%, rate of retention of mothers at T2 follow-up is ≥ 80% and the proportion of missing data in each completed questionnaire is ≤ 10%.

### Dissemination

The protocol has been completed as part of a PhD study conducted within the School of Nursing and Midwifery at Queens University, Belfast, Northern Ireland. Findings will be disseminated via academic audiences, relevant stakeholders and service users and social media.

## Discussion

To our knowledge, this study is the first of its kind to investigate the feasibility of a face-to-face group community-based intervention comprising music and social support for postnatal mothers and their infants. By offering this combined intervention to mothers in the early postnatal period in a group setting, we hope to establish its feasibility for a full-scale trial and promise of effect on outcomes including anxiety and stress. Acknowledging the known effect of poor postnatal mental health on mother-infant attachment and the desired addition of a social opportunity expressed by mothers in a previous process evaluation of a music intervention [22], it is intended that Mamas in Harmony will provide an accessible, supportive and calming environment for mothers to thrive.

## Supplementary Information

Supplementary Material 1: Appendix 1. Consent forms

Supplementary Material 2: Appendix 2. SPIRIT 2013 checklist: Recommended items to address in a clinical trial protocol and related documents

## Data Availability

Not applicable, the manuscript does not contain any data.

## Abbreviations

RCT: Randomised controlled trial
HSC: Health and social care
GAD- 7: Generalised anxiety disorder scale 7
EPDS: Edinburgh Postnatal Depression Scale
PSS: Perceived Stress Scale
KPCS: Karitane Parenting Confidence Scale
SSS: Social support survey
MPAS: Maternal Postnatal Attachment Scale
QUB: Queen’s University Belfast

## References

[1] Dennis C-L, Falah-Hassani K, Shiri R. Prevalence of antenatal and postnatal anxiety: Systematic review and meta-analysis. Br J Psychiatry. 2017;210(5):315–23. 28302701 10.1192/bjp.bp.116.187179

[2] NICE. Antenatal and postnatal mental health: clinical management and service guidance: NICE; 2020 [Available from: https://www.nice.org.uk/guidance/cg192/chapter/recommendations#anxiety-disorders. 31990493

[3] (RCOG) RCoOaG. Management of women with mental health issues during pregnancy and the postnatal period. 2011.

[4] Harrison S, Quigley MA, Fellmeth G, Stein A, Alderdice F. The impact of the Covid-19 pandemic on postnatal depression: analysis of three population-based national maternity surveys in England (2014–2020). Lancet Reg Health Eur. 2023;30:100654. 37363795 10.1016/j.lanepe.2023.100654 PMC10183799

[5] McMahon CA, Barnett B, Kowalenko NM, Tennant CC. Maternal attachment state of mind moderates the impact of postnatal depression on infant attachment. J Child Psychol Psychiatry. 2006;47(7):660–9. 16790001 10.1111/j.1469-7610.2005.01547.x

[6] Rossen L, Hutchinson D, Wilson J, Burns L, C AO, Allsop S, et al. Predictors of postnatal mother-infant bonding: the role of antenatal bonding, maternal substance use and mental health. Arch Women Ment Health. 2016;19(4):609–22. 10.1007/s00737-016-0602-z 26867547

[7] Holt C, Gentilleau C, Gemmill AW, Milgrom J. Improving the mother-infant relationship following postnatal depression: a randomised controlled trial of a brief intervention (HUGS). Arch Women Ment Health. 2021;19:19. 10.1007/s00737-021-01116-5 33742282

[8] Brummelte S, Galea LA. Postpartum depression: etiology, treatment and consequences for maternal care. Horm Behav. 2016;77:153–66. 26319224 10.1016/j.yhbeh.2015.08.008

[9] Beebe B, Steele M, Jaffe J, Buck KA, Chen H, Cohen P, et al. Maternal anxiety symptoms and mother-infant self- and interactive contingency. Infant Ment Health J. 2011;32(2):174–206. 25983359 10.1002/imhj.20274 PMC4431701

[10] NICE. Maternity and mental health: NICE; 2022 [Available from: https://www.nice.org.uk/about/what-we-do/into-practice/measuring-the-use-of-nice-guidance/impact-of-our-guidance/niceimpact-maternity/ch2-maternity-and-mental-health.

[11] Fawcett EJ, Fairbrother N, Cox ML, White IR, Fawcett JM. The prevalence of anxiety disorders during pregnancy and the postpartum period: a multivariate Bayesian meta-analysis. J Clin Psychiatry. 2019;80(4):1181. 10.4088/JCP.18r12527 PMC6839961 31347796

[12] Silverwood VA, Bullock L, Turner K, Chew-Graham CA, Kingstone T. The approach to managing perinatal anxiety: a mini-review. Front Psych. 2022;13:1022459. 10.3389/fpsyt.2022.1022459 PMC9797985 36590629

[13] Coates R, de Visser R, Ayers S. Not identifying with postnatal depression: a qualitative study of women’s postnatal symptoms of distress and need for support. J Psychosom Obstet Gynaecol. 2015;36(3):114–21. 26135567 10.3109/0167482X.2015.1059418

[14] Yang W-j, Bai Y-m, Qin L, Xu X-l, Bao K-f, Xiao J-l, et al. The effectiveness of music therapy for postpartum depression: a systematic review and meta-analysis. Complement Ther Clin Pract. 2019;37:93–101. 10.1016/j.ctcp.2019.09.002 31541788

[15] Morrell CJ, Warner R, Slade P, Dixon S, Walters S, Paley G, et al. Psychological interventions for postnatal depression: cluster randomised trial and economic evaluation. The PoNDER trial Health Technol Assess. 2009;13(30):1–153. 10.3310/hta13300 19555590

[16] Cooper PJ, Murray L, Wilson A, Romaniuk H. Controlled trial of the short- and long-term effect of psychological treatment of post-partum depression: I. Impact on maternal mood. Br J Psychiatry. 2003;182(5):412–9. 12724244

[17] Williams E, Dingle GA, Clift S. A systematic review of mental health and wellbeing outcomes of group singing for adults with a mental health condition. Eur J Pub Health. 2018;28(6):1035–42. 29982515 10.1093/eurpub/cky115

[18] Colella C, Lynn F, McNeill J. The effect of mother-infant group music interventions on postnatal depression – a systematic review. 2021. 10.1371/journal.pone.0273669 PMC9536550 36201504

[19] Fancourt D, Perkins R. Effect of singing interventions on symptoms of postnatal depression: three-arm randomised controlled trial. Br J Psychiatry. 2018;212(2):119–21. 29436333 10.1192/bjp.2017.29

[20] Ericksen J, Loughlin E, Holt C, Rose N, Hartley E, Buultjens M, et al. A therapeutic playgroup for depressed mothers and their infants: feasibility study and pilot randomized trial of community hugs. Infant Ment Health J. 2018;39(4):396–409. 29953626 10.1002/imhj.21723

[21] Perkins R, Yorke S, Fancourt D. How group singing facilitates recovery from the symptoms of postnatal depression: a comparative qualitative study. BMC Psychol. 2018;6(1):41. 30119704 10.1186/s40359-018-0253-0 PMC6098577

[22] Fancourt D, Perkins R. Creative interventions for symptoms of postnatal depression: a process evaluation of implementation. Arts Health. 2019;11(1):38–53. 31038038 10.1080/17533015.2017.1413398

[23] Bind R, Estevao C, Sawyer K, Rebecchini L, Hazelgrove K, Miller C, et al. Feasibility, clinical efficacy, and wellbeing outcomes of an online singing intervention for postnatal depression in the UK: SHAPER-PNDO, a single-arm clinical trial. Psychoneuroendocrinology. 2024;160:106910. 10.1186/s40814-023-01360-9 PMC10373337 37501172

[24] Warran K, Smith C, Ugron H, Blaga O, Ladegaard NL, Carstens LF, et al. Implementing a singing-based intervention for postpartum depression in Denmark and Romania: a brief research report. Front Med. 2023;10:1249503. 10.3389/fmed.2023.1249503 PMC10769490 38188326

[25] Estevao C, Bind R, Fancourt D, Sawyer K, Dazzan P, Sevdalis N, et al. SHAPER-PND trial: clinical effectiveness protocol of a community singing intervention for postnatal depression. BMJ Open. 2021;11(11):e052133. 34789494 10.1136/bmjopen-2021-052133 PMC8601068

[26] Chan AW, Tetzlaff JM, Altman DG, Laupacis A, Gøtzsche PC, Krleža-Jerić K, et al. SPIRIT 2013 statement: defining standard protocol items for clinical trials. Ann Intern Med. 2013;158(3):200–7. 23295957 10.7326/0003-4819-158-3-201302050-00583 PMC5114123

[27] Eldridge SM, Chan CL, Campbell MJ, Bond CM, Hopewell S, Thabane L, Lancaster GA. CONSORT 2010 statement: extension to randomised pilot and feasibility trials. BMJ. 2016;355:i5239. 10.1136/bmj.i5239. https://www.bmj.com/content/355/bmj.i5239 10.1136/bmj.i5239 PMC5076380 27777223

[28] DHSSPS. Healthy Child, Health Future. A Framework for the Universal Child Health Promotion Programme in Northern Ireland. 2010.

[29] Spitzer RL, Kroenke K, Williams JB, Löwe B. A brief measure for assessing generalized anxiety disorder: the GAD-7. Arch Intern Med. 2006;166(10):1092–7. 16717171 10.1001/archinte.166.10.1092

[30] Cox JL, Holden JM, Sagovsky R. Detection of postnatal depression. Development of the 10-item Edinburgh Postnatal Depression Scale. Br J Psychiatry. 1987;150:782–6. 10.1192/bjp.150.6.782 3651732

[31] Cohen S, Kamarck T, Mermelstein R. A global measure of perceived stress. J Health Soc Behav. 1983;24(4):385–96. 10.2307/2136404. 6668417

[32] Črnčec R, Barnett B, Matthey S. Development of an instrument to assess perceived self-efficacy in the parents of infants. Res Nurs Health. 2008;31(5):442–53. 18297638 10.1002/nur.20271

[33] Sherbourne CD, Stewart AL. The MOS social support survey. Soc Sci Med. 1991;32(6):705–14. 2035047 10.1016/0277-9536(91)90150-b

[34] Condon JT, Corkindale CJ. The assessment of parent-to-infant attachment: development of a self-report questionnaire instrument. J Reprod Infant Psychol. 1998;16(1):57–76.

[35] Herdman M, Gudex C, Lloyd A, Janssen M, Kind P, Parkin D, et al. Development and preliminary testing of the new five-level version of EQ-5D (EQ-5D-5L). Qual Life Res. 2011;20(10):1727–36. 21479777 10.1007/s11136-011-9903-x PMC3220807

[36] Lewis M, Bromley K, Sutton CJ, McCray G, Myers HL, Lancaster GA. Determining sample size for progression criteria for pragmatic pilot RCTs: the hypothesis test strikes back! Pilot Feasib Stud. 2021;7:1–14. 10.1186/s40814-021-00770-x PMC7856754 33536076

[37] Green J, Thorogood N. Qualitative Methods for Health Research. 4th ed. London: SAGE; 2018.

[38] QUB. Data Protection: Queen’s University Belfast; 2023 [Available from: https://www.qub.ac.uk/about/Leadership-and-structure/Registrars-Office/Information-Compliance-Unit/Data-Protection/.

[39] Schulz KF, Altman DG, Moher D, the CG. CONSORT 2010 statement: updated guidelines for reporting parallel group randomised trials. Trials. 2010;11(1):32. 10.4103/0976-500X.72352 PMC3043330 21350618

[40] Braun V, Clarke V, Terry G, Rohleder P, Lyons A. Qualitative research in clinical and health psychology. London, England: Palgrave Macmillan; 2014.

[41] Campbell KA, Orr E, Durepos P, Nguyen L, Li L, Whitmore C, et al. Reflexive thematic analysis for applied qualitative health research. Qualitat Rep. 2021;26(6):2011–28.

