# Supplementary material for: Mamas in Harmony: protocol for a pilot RCT and process evaluation of a music and social support intervention establishing the feasibility of reducing anxiety and stress in postnatal mothers
Source: Pilot Feasibility Stud. 2025 Apr 10;11:44. doi: 10.1186/s40814-025-01629-1 (PMC11984032; doi:10.1186/s40814-025-01629-1)
Supplement: Supplementary file 1 — Supplementary Material 1: Appendix 1. Consent forms [file 40814_2025_1629_MOESM1_ESM.docx]

| **Consent form for Mamas in Harmony study**  STUDY ID __________ | **Please initial** |
| --- | --- |
| 1. I confirm that I have been given and have read and understand the Participant Information Sheet (15-01-2023 Version 1.1). I have had the opportunity to ask and receive answers to any questions I may have had. |  |
| 1. I understand that my participation is voluntary and that I am free to withdraw at any time, without giving any reason and without my legal rights being affected. |  |
| 1. I understand that the data provided until the point of withdrawal will be retained and used for analysis. |  |
| 1. I understand that my personal information will be held securely on university premises and should comply with relevant data protection legislation and data collected as part of the research may be looked at by authorised individuals from Queen’s University Belfast (QUB) where relevant and I give permission for these individuals to have access to this information. |  |
| 1. I understand what is discussed during the Mamas in Harmony study will be kept confidential by the research team with the exception that if I disclose information that indicates that I or someone I mention is at risk of harm, the researcher is legally obliged to pass on this information in accordance with professional guidelines. 2. The research team cannot ensure confidentiality of information shared between, and/or by other mothers during or after Mamas in Harmony sessions |  |
| 1. I understand that I will not be identifiable in any data published in relation to this project. 2. I agree to take part in the above study. |  |

___________________ _____________ _____________________

Name of Participant Date Signature

___________________ _____________ _____________________

Researcher Date Signature

**Consent form for interview for mothers**

| STUDY ID __________ | **Please initial** |
| --- | --- |
| 1. I confirm that I have been given and have read and understand the Participant Information Sheet (21-08-2022 Version 1.0) for the above interview. I have had the opportunity to ask and receive answers to any questions I may have had. |  |
| 1. I understand that my participation is voluntary and that I am free to withdraw at any time, without giving any reason and without my legal rights being affected. |  |
| 1. I understand that the data provided until the point of withdrawal will be retained and used for analysis. |  |
| 1. I understand the interview is being conducted by a researcher from Queen’s University Belfast (QUB) and that my personal information will be held securely on university premises and handled in compliance with Data Protection legislation, and data collected as part of the research may be looked at by authorised individuals from QUB where relevant and I give permission for these individuals to have access to this information. |  |
| 1. I understand that what is discussed during the interview is confidential with the exception that if I disclose information that indicates that I or someone I mention is at risk of harm, the researcher is legally obliged to pass on this information in accordance with professional guidelines. 2. I understand that the interview will be digitally recorded if using Microsoft Teams or audio recorded if completed in person face to face. The recording will then be deleted once transcribed. 3. I understand direct quotes may be used in papers or reports but that these quotes will not be identifiable |  |
| 1. I understand that I will not be identifiable in any data published in relation to this project. 2. I agree to take part in the above interview. |  |

______________________ _____________ _____________________

Name of Participant Date Signature

________________________ _____________ _____________________

Researcher Date Signature

**Consent form for interview of intervention facilitators**

|  | **Please initial** |
| --- | --- |
| 1. I confirm that I have been given and have read and understand the Participant Information Sheet (15-01-2023 Version 1.1) for the above interview. I have had the opportunity to ask and receive answers to any questions I may have had. |  |
| 1. I understand that my participation is voluntary and that I am free to withdraw at any time, without giving any reason and without my legal rights being affected. |  |
| 1. I understand that the data provided until the point of withdrawal will be retained and used for analysis. |  |
| 1. I understand the interview is being conducted by researchers from Queen’s University Belfast (QUB) and that my personal information will be held securely on university premises and should comply with relevant data protection legislation and data collected as part of the research may be looked at by authorised individuals from QUB where relevant and I give permission for these individuals to have access to this information. |  |
| 1. I understand that what is discussed during the interview is confidential with the exception that if I disclose information that indicates that I or someone I mention is at risk of harm, the researcher is legally obliged to pass on this information in accordance with professional guidelines. |  |
| 1. I understand that the interview will be digitally recorded if using Microsoft Teams or audio recorded if completed in person face to face. The recording will then be deleted once transcribed. |  |
| 1. I understand direct quotes may be used in papers or reports but that these quotes will not be identifiable |  |
| 1. I understand that I will not be identifiable in any data published in relation to this project. |  |
| 1. I agree to take part in the above interview |  |

______________________ _____________ _____________________

Name of Participant Date Signature

________________________ _____________ _____________________

Researcher Date Signature
